# Coulomb-driven single defect engineering for scalable qubits and spin sensors in diamond

**DOI:** 10.1038/s41467-019-12556-0

**Published:** 2019-10-31

**Authors:** Tobias Lühmann, Roger John, Ralf Wunderlich, Jan Meijer, Sébastien Pezzagna

**Affiliations:** Applied Quantum Systems, Felix-Bloch Institute for Solid-State Physics, University Leipzig, Linnéstraße 5, 04103 Leipzig, Germany

**Keywords:** Quantum information, Optical properties of diamond, Condensed-matter physics

## Abstract

Qubits based on colour centres in diamond became a prominent system for solid-state quantum information processing and sensing. But the deterministic creation of qubits and the control of their environment are still critical issues, preventing the development of a room-temperature quantum computer. We report on the high creation yield of NV centres of 75% (a tenfold enhancement) by charge-assisted defect engineering, together with an improvement of their spin coherence. The method strongly favours the formation and negative charge state of the NV centres with respect to intrinsic diamond, while it hinders the formation of competing and perturbing defects such as di-vacancies or NVH complexes. We evidence spectrally the charge state tuning of the implantation-induced vacancies from V^0^ to V^−^, key element of this Coulomb-driven engineering. The generality of the method is demonstrated using several donors (phosphorous, oxygen and sulphur) and applying it to other centres (SnV and MgV) in diamond.

## Abstract (web summary)

Nitrogen vacancy centres occur naturally in diamond and have potential uses in quantum computing but many applications require scalable, accurate fabrication methods. Here the authors demonstrate that tuning the doping modifies the dynamics of centre formation, increasing yields and coherence times.

## Introduction

Qubits in diamond based on the nitrogen-vacancy (NV) centre are widely implemented for a large range of applications like quantum information processing^1^, quantum sensing of magnetic fields^2^, electric fields^3^, or temperature^4^ but also for nuclear magnetic resonance^5^ or even to answer fundamental questions in physics^6,7^. The fabrication and reproducibility of scalable devices requires (and still lacks) sample homogeneity and creation yields of the qubits close to 100%^8^, but as well, the control and engineering of their environment^9^. The addressing using ion implantation provides high spatial resolution^10^, placement accuracy^11^, and even deterministic countable single ions^12–15^. However, the formation of impurity-vacancy centres (NV, SiV, SnV,…) using a thermal treatment^16,17^ enabling the mobility of the implantation-induced vacancies^18,19^ is a statistical process of generally low efficiency (a few % or less^20^). It is known that a large part of these vacancies are lost by recombination with carbon interstitials, by formation of di-vacancies or other complexes (vacancy chains, VH) or by diffusion to dislocations or to the diamond surface for the shallow ones. It was moreover shown that the proportion of vacancy complexes (with respect to isolated vacancies) increases with the atomic mass of the implanted ions^9^ and that suitable thermal treatments enable the dissociation of vacancy chains^21^. Efforts were also made to increase the NV yield (for example by co-implantation of carbon^22^, post-irradiation with electrons or protons to produce additional vacancies^23,24^ or more recently very efficiently by laser writing^25^) and to understand the NV formation process^26,27^. Not only for scalability purposes, high creation yields would ensure a clean environment free of unconverted and perturbing atoms, for example, nitrogen in the case of NV centres^28^, whereas a close-by donor (such as nitrogen or phosphorous) is necessary to keep the charge state stability^29–32^ necessary for most applications.

Furthermore, hydrogen is by orders of magnitude the most abundant impurity in ultrapure IIa CVD diamonds (owing to the hydrogen plasma assisted growth) but its exact concentration and role in colour centre formation are difficult to evaluate. Though, it was shown that the NVH concentration in CVD grown layers is typically at least one order of magnitude larger than the NV centres concentration, whatever the nitrogen content^33^, and that the formation energy of the NVH defect is ~1 eV lower than that of the NV centre^28^. The low yields of implanted NV centres may also be owing to hydrogen diffusion and NV passivation^9,34,35^ during the thermal treatments. Note that besides temperature^34^, hydrogen (deuterium) diffusion is known to also strongly depend on defect concentration, doping^36,37^, or electric bias^38^.

Recently, using a sacrificial B-doped diamond layer led to a better formation yield of shallow NV centres (from 1 to 2%)^39^. This was attributed to the charging of single vacancies (otherwise mostly neutral in intrinsic diamond), which was expected to hinder the formation of di-vacancies. Also, the assumption that the chemical potential is pinned down enough to charge vacancies positively would then imply that substitutional nitrogen atoms are also positively charged, which may eventually hinder the formation of the NV centres (if one agrees with the previous electric repulsion argument leading to less di-vacancies). Ab initio calculations of the adiabatic charge transition levels of V and N can be found in^27^.

On the other hand, we intend to charge the vacancies negatively V^−^, whereas having positively charged nitrogen N^+^, which may enhance the NV centre formation even more (still preventing V-V formation) using Coulomb forces. This is one of the main questions addressed in this work. Such a situation is likely accessible using a suitable n-type doping and other questions we address are whether using phosphorous implantation can provide enough electrically active donors (phosphorous-doped CVD grown (100) diamond is generally significantly compensated^40,41^ owing to the presence of acceptor defects), how alternative donors compare with phosphorous^42,43^, and how the qubits coherence time compares with intrinsic diamond.

## Results

### Evidence of vacancy charging by efficient phosphorous doping

**Fig. 1:**
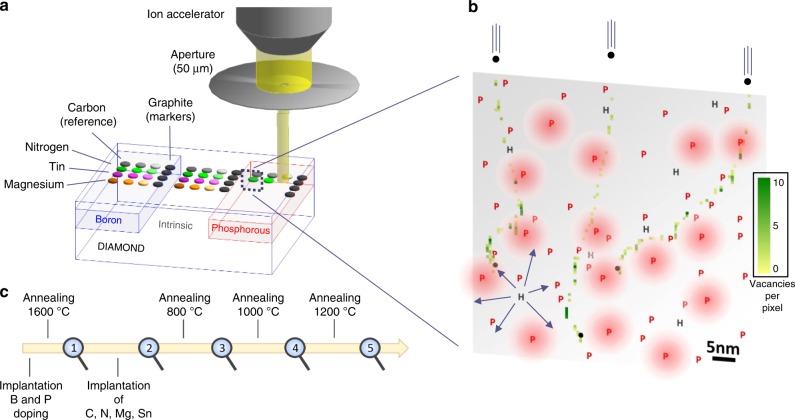
Colour centre implantation within three doped regions. **a** Scheme of the diamond sample (quantum grade from Element 6) prepared with intrinsic, p-type and n-type areas (using boron and phosphorous implantation with [P] and [B]  ~  3 × 10^18^ cm^−3^, see supplementary information) and implanted with a series of different chemical elements: carbon (as reference) and nitrogen, tin, and magnesium to produce, NV, SnV and MgV centres. The depth of the centres (~ 50 nm) is controlled by the implantation energy and fits with the dopant depth profiles (see supplementary information). **b** Illustration of the sample status after implantation in the Phosphorous-doped region, as represented by a 60 × 60 nm² cross-section. The implantation-induced vacancies are simulated by SRIM. The density is given in number of vacancies per pixel of size 0.6 × 0.6 nm². The phosphorous atoms are represented by the red P letters, with inter-distance corresponding to [P] ≈ 3 × 10^18^ cm^−3^. The red halos represent substitutional P active donors which are non-passivated and non-self-compensated. Hydrogen atoms are also represented at a density of 1 × 10^17^ cm^−3^, with arrows accounting for their large diffusion (~ 6 × 10^7^ nm² s^−1^ at 800 °C in IIa diamond^34^). Less than 2.5 nitrogen and 0.5 boron atoms are expected within this section for native concentration [N] < 5 ppb and [B] < 1 ppb. NVH, V_2_, V_x_, VH, and PV are the main competing defects to NV formation, whereas PH may passivate the donors. NVN forms at much higher temperatures and/or [N] densities owing to the low diffusion of nitrogen (~ 1.7 × 10^−3^ nm².s^−1^ at 1600 °C in ref. ^9^). **c** Time sequence of the different steps of the study. The magnifiers symbolise imaging and spectroscopy

**Fig. 2:**
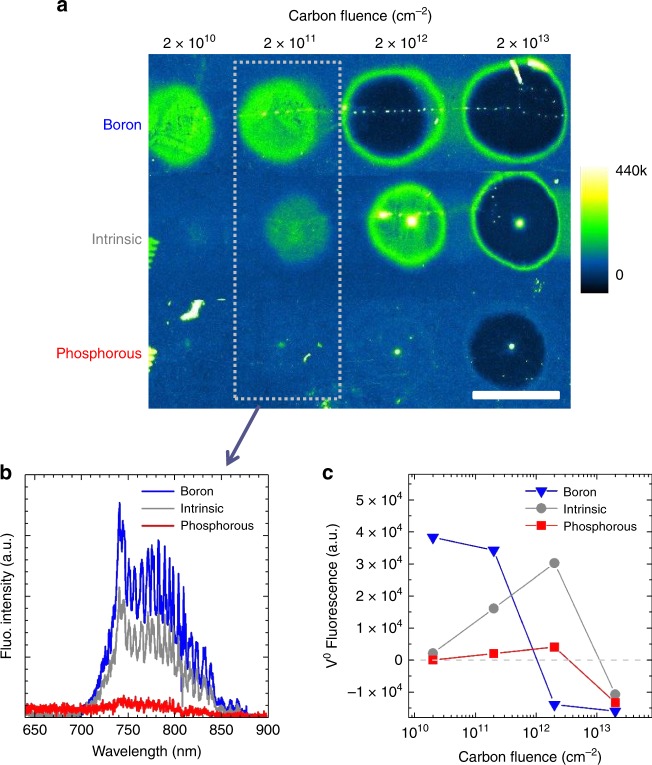
Charge state tuning of single vacancies by diamond doping. **a** Confocal fluorescence scans of the carbon implantations with fluences in the range 2 × 10^10^ – 2 × 10^13^ cm^−2^, repeated within the three different doped regions: p-type, intrinsic and n-type with [B] and [P] ~ 3 × 10^18^ cm^−3^. The excitation wavelength is 532 nm and the detection is above 650 nm to record the signal from neutral vacancies V^0^ (GR1 centre, ZPL at 741 nm). The scale bar is 50 µm. The bright dots visible in some implanted spots are owing to a focused electron beam during cathodoluminescence measurements (see supplementary Figure 11). **b** Fluorescence spectra recorded in the three doped areas for the 2 × 10^11^ cm^−2^ carbon fluence (acquisition by scanning a 10 × 10 µm² area at the centre of each spot). The characteristic emission from the GR1 is recognised. The absence of GR1 fluorescence in the P-doped area is owing to charging up of the vacancies in their negative state V^−^. The charge state transition levels of vacancies can be found in^27^ **c** Average V^0^ fluorescence intensity (with respect to the background fluorescence level given as the zero level) as a function of the carbon fluence and diamond doping

We study the creation efficiency, thermal stability, and charge states of implanted NV, SnV, and Mg-related centres as a function of the doping level of diamond, using the most commonly employed acceptor (boron, *E*_a_ = 0.37 eV) and donor (phosphorous, *E*_d_ = 0.57 eV) and in a second step alternative donors (oxygen, *E*_d_ = 0.32 eV^42^ and Sulphur, *E*_d_ = 0.77 eV^43^). We firstly give evidence of the control of the charge state of single vacancies after implantation (and before thermal annealing), which we then systematically correlate to the formation efficiency of the different colour centres. The methodology consists in a sequence of ion implantations (doping and colour centres), thermal annealings and optical and spin characterisations using fluorescence confocal microscopy, as resumed in Fig. 1. The preliminary experiment consists in the implantation of carbon ions, repeated into the different doped areas in order to produce vacancies, measure their charge state (before thermal annealing) and evaluate the efficiency of the phosphorous and boron doping. For this, we follow (as in Fig. 2b) the characteristic optical fluorescence of isolated neutral vacancies V^0^ with zero-phonon-line (ZPL) at 741 nm^17^. The main result here is that Phosphorous doping induces a negative charging of the vacancies into V^−^ (the absorption band of negatively charged vacancies V^−^ with ZPL at 394 nm (ND1)^17^ cannot be observed here and does not contribute any fluorescence). Note that, as shown by Collins^44^, the charge state of a defect centre in diamond is depending on the presence of a close-by donor^31^ or acceptor rather than the position of the chemical potential, which is moreover difficult to calculate in an insulating material under light illumination. Here, the absence of V^0^ fluorescence means therefore that there are enough electrically active phosphorous atoms to provide electrons to the single vacancies, or in a semiconductor view, to pin up the chemical potential above the V(0/–) charge transition of the single vacancies. As seen in Fig. 2, the V^0^ fluorescence from the C implantation is strengthened in the B-doped area whereas it is suppressed in the P-area (fully tuned to V^−^) with respect to intrinsic diamond. For the highest fluence of 2 × 10^13^ cm^−2^ (close to the amorphisation threshold), the implantation damage results in a lower fluorescence than the background of the unimplanted diamond. In the B-doped region however, this higher damage already occurs for the 2 × 10^12^ cm^−2^ fluence. These damaged areas reveal the formation of vacancy complexes or even voids, also predicted by molecular dynamics simulations^9^. Their occurrence at lower fluences in the B-doped region is owing to the fact that in this region, where the V^0^ charge state is strengthened, the isolated vacancies can group together more easily. By opposition, in the P-doped area where the vacancies are negatively charged, the high damage is only visible at a 2 × 10^13^ cm^−2^ fluence, revealing that vacancy clustering is reduced. The intrinsic region presents an intermediate situation for which both V^0^ and V^−^ are present, indicating that the chemical potential lies close to the V(0/–) transition level during the measurement (under laser excitation). Note that no fluorescence is known from the positively charged vacancy V^+^. These results confirm that isolated vacancies are preserved when they are charged, whereas vacancy complexes can form more easily when they are neutral. The consequences of this on the impurity-vacancy defects formation will be discussed in the next sections.

Note that an estimation of the efficiency of implanted phosphorous as an active donor (in substitutional site, non-passivated, and non-compensated) by comparing the number of created vacancies using SRIM simulations^45^ and the number of phosphorous atoms previously implanted indicates that the phosphorous atoms are efficiently placed in substitutional sites and that the formation of complexes such as PH or PV (passivating or compensating the donor, respectively)^46,47^ is likely low. An occupation of substitutional sites of 70% by implanted phosphorous after thermal annealing was measured using internal channelling measurements^48^. Recently, single optically active defects likely related to phosphorous were reported in diamond with a creation yield of 10^−4^ to 10^−5^^9^ but of still unknown structure.

### NV centres: doping-dependent creation efficiency

**Fig. 3:**
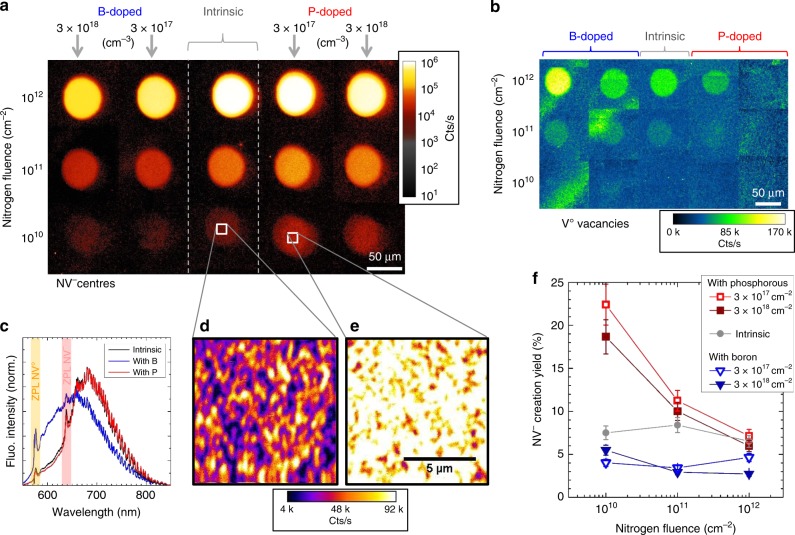
Doping-dependent NV centre formation at 800 °C. **a** Confocal fluorescence scans of the nitrogen implantations in the different doping areas recorded after thermal annealing at 800 °C for 4 h. The laser excitation is 532 nm and a longpass filter 600 nm is used to record the NV centres fluorescence. The fluorescence intensity is in logarithmic scale. **b** Confocal fluorescence scans of the same areas measured directly after nitrogen implantation and before annealing. A longpass filter 700 nm is used to record V^0^ (GR1) fluorescence. **c** Normalised fluorescence spectra of the NV centres fluorescence (10^12^ cm^−2^ fluence) showing the effect of the boron and the phosphorous doping with respect to the intrinsic region, respectively. The ZPL of NV^0^ (575 nm) and of NV^−^ (638 nm) are highlighted. Contrarily to boron (giving more NV^0^), the effect of phosphorous on the NV charge state is minimal because for a 7.5% yield there are already ~ 13 unconverted N donors for each NV centre. **d** and **e** are closer scans (with linear fluorescence scale) of two regions of interest of low nitrogen fluence (10^10^ cm^−2^) for the intrinsic and phosphorous-doped diamond ([P] ~ 3 × 10^17^ cm^−3^). **f** NV centres creation yield vs nitrogen fluence and doping type/level, deduced from **a**, **d**, and **e**. The Phosphorous doping induces up to a threefold yield improvement from 7.5% to 22.4%. The error bars represent the measurement uncertainties derived from the yield formula given in supplementary Equation 3

Before annealing, the fluorescence of vacancies induced by nitrogen implantation (Fig. 3b) reveals, as for carbon atoms, that the vacancies are fully tuned to V^−^ when the phosphorous doping is increased, whereas V^0^ fluorescence is clearly visible in the intrinsic and the boron-doped areas. The pre-annealing situation is illustrated in Fig. 1b, with average distances between impurities given at scale (and extrapolated for a 2D cross-section) for a nitrogen fluence of 1 × 10^11^ cm^−2^ and concentrations [P] ≈ 12 ppm, [H] ≈ 600 ppb, [N] < 5 ppb and [B] < 1 ppb. Note that the donor effect of nitrogen itself can be neglected here because ~ 160 vacancies are created for each implanted nitrogen.

Before the 800 °C annealing step is conducted, the vacancies thus have different charge states depending on the doped area. The nitrogen atoms, when placed in substitutional sites, are expected to be in N^+^ state (vacancies are compensating acceptors for nitrogen and in large majority before annealing, Fig. 2c (see supplementary Figure 10)). The consequence of this can now be evaluated from the NV centres fluorescence in Fig. 3a after 800 °C annealing. The NV creation yield is calculated for each implantation spot and plotted in Fig. 3f. For intrinsic diamond, it is in the range 6–8%, which is typical for this implantation energy^9,20,22^ (and the same for either as-grown or polished diamond). Interestingly, the B- and P-doping give rise to very different behaviours: the creation yield is reduced for B-doping (about twice lower), whereas it is enhanced for P-doping. The highest effect of phosphorous is observed in the low nitrogen fluence regime, for which the ratio “dopants over other defects” is maximum. The NV yield has a threefold increase up to 22.4% under the effect of phosphorous, as also highlighted by Fig. 3d, e. This corresponds to the ideal situation during thermal annealing for which the involved impurities are mostly in the following charge states: V^−^, N^+^, P^+^, and H^−^ (hydrogen in the bond-centred site has acceptor behaviour with H(0/–) ~ 2 eV below the conduction band^49^), thus likely hindering the formation of competing NVH, V_2_, and VH defects, in favour of NV. Note that the slightly enhanced NV yield observed in reference^39^ using boron doping is owing to the high doping level ([N_A_] = 10^20^ cm^−3^), which likely induces a V^+^ charge state precluding the formation of di-vacancies, whereas the implanted boron concentration used here (2 × 10^18^ cm^−3^) stabilises V^0^, thus leading to more di-vacancies and a lower NV yield.

### Tin-vacancy and Mg-related centres

**Fig. 4:**
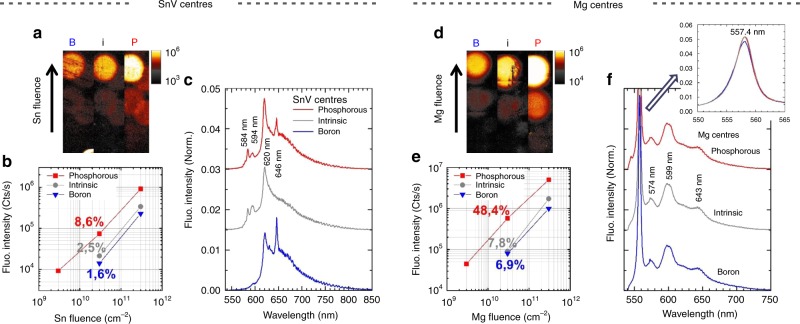
Doping-dependent SnV and MgV centre formation at 1200 °C. **a** Confocal fluorescence scans of the SnV centres implanted in the different doping areas, recorded after the last thermal annealing at 1200 °C for 4 h. The fluorescence intensity is in logarithmic scale. The laser excitation is 532 nm and the detection with a longpass filter 550 nm. **b** Plot of the fluorescence intensity vs fluence, for the different doping conditions. The corresponding creation yields are indicated. **c** Normalised fluorescence spectra measured from the different Sn-implanted areas. The spectra are recorded within the 10 × 10 µm² central region of each implanted spot. The spectra are background corrected (with references taken from 10 × 10 µm² unimplanted neighbouring areas). **d**, **e**, and **f**, same for Mg-related centres

This Coulomb-driven defect engineering has also been applied to SnV and Mg-related^9^ centres to establish whether the previous results are specific to NV centres or possibly a more general trend for impurity-vacancy centres. Recently, it was shown that high yields of SiV^0^ centres were obtained in boron-doped diamond^50^. Our method (Fig. 1a) allows the direct comparison of different impurity-vacancy defects in exactly the same conditions: same diamond sample, same doping levels, same thermal annealing and same measurement parameters, at the exception of possible spatial inhomogeneity within the sample. The properties of column IV—vacancy (SiV, GeV, SnV, and PbV) centres arouse growing interest^51–54^ and the creation of single SnV centres was reported recently, based on Sn implantation and thermal annealing^51,52^. Creation yields of implanted SnV centres were reported in the range 1–5% (in undoped diamond), comparable to what is obtained for SiV^55^ or GeV^56^. Owing to the high damage induced by the large Sn atom^9^, higher temperatures (also using high pressure to prevent graphitisation) were preferred, which indeed led to reduced ZPL width^52^ (indicative of a less defected environment) and to the removal of the fluorescence peaks at 595 nm and 646 nm observed for temperatures below 1500 °C. Figure 4a is a fluorescence plot of the Sn implantation spots, after the temperature step at 1200 °C. The corresponding spectra in Fig. 4c confirm the formation of SnV, with the characteristic ZPL at 620.3 nm. The main result is that the highest SnV fluorescence is found in the phosphorous region (Fig. 4b), with a creation yield of 8.6%, which is more than three times larger than in the intrinsic (2.5%) and the B-doped (1.6%) areas. These results reveal the same behaviour as what was obtained for the NV centres, which can be explained by a more efficient formation of SnV together with a reduced formation of competing centres. Note that unlike the NV, the structure of the SnV centre has D_3d_ symmetry (like the SiV and GeV), with the Sn atom sitting between two vacancies in an interstitial position. A discussion about the possible charge states of the SnV centre is found in the supplementary Figure 9.

Concerning the Mg-related centres, which are believed to involve vacancies (because single centres exhibit a strong polarisation anisotropy in excitation, see supplementary Figure 8), the fluorescence measured from the implantation spots in Fig. 4d shows the characteristic emission at 557.4 nm^9^ (Fig. 4f). The corresponding charge state is still unknown. No doping dependence is observed in the MgV spectra (between 550 nm and 900 nm and at all temperatures) at the exception of the weak feature at 544.5 nm in the P-doped region). Like in the case of the NV and SnV centres, the Mg-centre emission is at strongest in the P-doped region, here with up to a sixfold improved yield (for the 3 × 10^10^ cm^−2^ Mg fluence) from 7.8% in the intrinsic area to 48.4% in the P-doped area (as seen in Fig. 4e). This result further supports the assumption of a vacancy involved in the centre. Note that it cannot be excluded here that a less negative state (than the 557.4 nm ZPL) of the MgV centre may be optically inactive (or above 900 nm) and lead to the higher yield of the 557.4 nm MgV. Further studies such as ab initio calculations or active electrical tuning^57^ of single centres are necessary.

### Temperature evolution

**Fig. 5:**
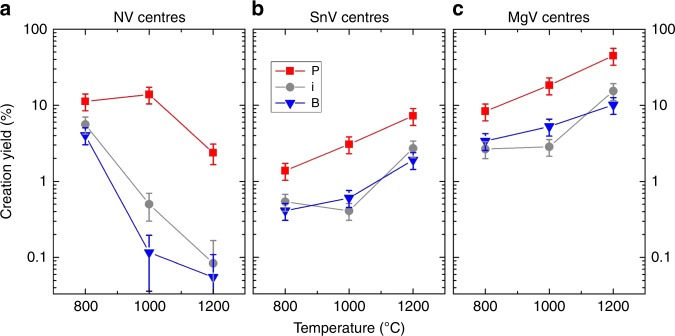
Temperature evolution of colour centre population. **a** Creation yield (ratio of NV density and implanted nitrogen fluence, here 3 × 10^11^ cm^−2^) of NV centres measured after the different consecutive annealing steps of 4 h each and for the different doping conditions. The effective yield enhancement between intrinsic and P-doped diamond is of three at 800 °C, whereas it is of ~ 27 at 1000 °C. **b** Same graph for SnV centres (Sn fluence of 3 × 10^11^ cm^−2^). **c** Same graph for MgV centres (Mg fluence of 3 × 10^11^ cm^−2^). The error bars represent the measurement uncertainties derived from the yield formula given in supplementary Equation 3

The temperature dependence of the centres density (Fig. 5) confirms the higher yields in phosphorous-doped diamond in all cases. However, although the SnV and MgV population keeps increasing with the annealing temperature, the NV centres behave differently. The NV yield drops strongly (more than one order of magnitude) for intrinsic and B-doping between 800 °C and 1000 °C, whereas it still increases (from 11 to 14%) in the P-doped area, before it finally decreases at 1200 °C. This striking difference cannot be explained by N diffusion and formation of NVN (H3 centres with ZPL at 503 nm), which was not measured here and is only significant for much higher nitrogen densities and/or temperatures^9^. NV dissociation is expected to occur at higher temperatures as it was shown that a temperature of 1150 °C (90 min) is not high (long) enough to induce a change of the NV direction (owing to vacancy diffusion)^9^. We attribute this precocious loss of NV centres in the intrinsic and boron-doped areas to their passivation by hydrogen^9,34,35^, knowing that the charge state and diffusion of hydrogen are doping-dependent^36,37^ and that the NV centres are preserved by the phosphorous doping.

These results indicate that, contrarily to SnV and MgV centres, a compromise is likely to be found for the optimal formation of NV centres upon thermal annealing, between forming NV centres, recovering a healed crystal structure without vacancy complexes^21^ and avoiding hydrogen passivation. The temperature range 900 °C–1100 °C seems to be adequate for diamond pre-doped with donors, but the hydrogen native concentration and the annealing dynamics and times are also important parameters.

### Oxygen and Sulphur as efficient donors

**Fig. 6:**
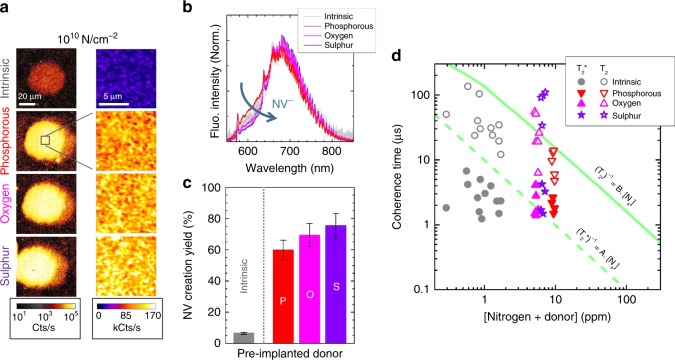
NV centres with different donors. **a** Confocal fluorescence scans of the NV centres created in the intrinsic and donor-doped areas (nitrogen fluence of 1 × 10^10^ cm^−2^, energy of 40 keV, annealing at 800 °C for 4 h). The different donors (phosphorous, oxygen, and sulphur) were pre-implanted and annealed at 1200 °C. The laser excitation wavelength is 532 nm and the detection window 600–700 nm. The fluorescence intensity is plotted in logarithmic scale. The insets are 10 × 10 µm² scans taken at the centre of each implanted spot, plotted in linear scale. **b** Fluorescence spectra (normalised) from the NV centres in the different areas. The NV^−^ charge state stabilisation increases from P to O and S. **c** NV creation yield vs pre-implanted donor species, together with intrinsic diamond. Donor pre-implantation leads to one order of magnitude increase of NV density and is highest for sulphur. The error bars represent the measurement uncertainties derived from the yield formula given in supplementary Equation 3. **d** Coherence times T_2_ and T_2_* of several single NV centres as a function of the sum of implanted nitrogen and donor concentration. The T_2_* and T_2_ times are obtained, respectively, from the decay of Ramsey (free induction decay) and Hahn echo sequences applied to the single NV centres (with same fit function used as in^39^). The lines account for the usually observed inverse proportionality between T_2_, T_2_* and the nitrogen concentration, with A = 101 ms^−1^/ppm and B = 6.5 ms^−1^/ppm^28^

Another sample was prepared as in Fig. 1, but with the additional donors oxygen and sulphur, and annealed at 1200 °C instead of 1600 °C. Nitrogen was then implanted and annealed at 800 °C for 4 h. The creation yield of NV centres (Fig. 6) increases of about one order of magnitude in the donor areas with respect to intrinsic diamond and reaches 69.3% for oxygen and 75.3% for Sulphur. The ensemble fluorescence spectra reveal that Sulphur induces also the most negative NV^−^ average charge state. For phosphorous, the reduced annealing temperature of 1200 °C used here gives a three times better yield (59.7%) than for the previous 1600 °C treatment. This can be due to an increased compensation and passivation of the donors at higher temperature, as observed for phosphorous-doped CVD grown layers when post-annealed at high temperature^40^.

Finally, the spin properties of the NV centres were studied in all areas and T_2_ and T_2_* coherence times of several NV centres are plotted in Fig. 6c, as a function of the concentration of implanted nitrogen and donors. The green lines represent the standard observed dependence of T_2_ and T_2_* on the nitrogen concentration for as-grown N-doped diamond^28^. In the intrinsic area, the coherence times are shorter, due to residual implantation defects, especially di-vacancies^21^. Among all donors, T_2_ times are at best in the sulphur area, indicating that the excess of (likely) neutral donors—about one NV centre for ten donors—leads to decoherence in the case of phosphorous but does not, or much less, in the case of sulphur. The coherence times are improved (reaching up to ~ 100 µs) with respect to an equivalent nitrogen concentration of about 7 ppm (typically 20 µs).

## Discussion

The method and results presented here show that the creation yields of NV and other defect centres are strongly dependent on the doping level of diamond, and that 75% are achieved using Sulphur doping. The donors modify the centres dynamics of formation during thermal annealing through the charge state and diffusion of the involved species, with a major role likely played by vacancies and hydrogen. The yield is also strongly dependent on the annealing temperature. The highest creations of NV, SnV, and MgV centres obtained in donor-doped diamond, for which isolated vacancies were shown to be negatively charged V^−^, indicates that these are preserved from forming vacancy complexes in profit of NV, SnV, or MgV. The preliminary carbon implantation also showed that in Boron-doped diamond, where the vacancies are kept neutral, vacancy clustering does take place at lower implantation fluence than in intrinsic or Phosphorous-doped diamond. Charge state tuning of the defects may also be achieved during implantation and/or annealing by applying electrical bias to the sample or using UV illumination.

Highly enhanced creation yields were demonstrated here: 75.3%, 8.6%, and 48.4% for NV, SnV, and MgV, respectively. Even if not yet fully deterministic, the scalability of NV^−^based devices is dramatically improved. As an example, to obtain a 5-qubit system with the implantation of five nitrogen atoms would require ~1 million attempts with a 6.5% yield, whereas only four attempts are needed with a 75% yield.

Besides, the coherence properties of the NV centres (T_2_ times) are improved in Sulphur-doped diamond, which moreover provides the highest NV^−^ stability. The clear correlation between charging of vacancies and improved creation yield of NV, SnV, and MgV centres shows that there is plenty of room on the route(s) towards scalable QIP diamond devices and that the deterministic creation of qubits centres may eventually be achieved. The developments of ion implantation techniques are approaching their limits, both in terms of spatial resolution (a few nm) and of number of ions, as different methods enable a priori or a posteriori the counting and deterministic implantation of single ions. Furthermore, colour centres can be optically imaged with a resolution below 10 nm with stimulated emission depletion microscopy. Obviously, diamond inhomogeneities and hydrogen impurities are among the main issues to be overcome in the future. Diamond (area) preselection methods will be developed and traps for hydrogen will for example be designed and implanted to capture the diffusing hydrogen atoms before they may reach and passivate the implanted qubits. Finally, this work gives original insights into *n*-type doping by ion implantation. The recent discovery of luminescent P-related centres together with new opportunities in defect engineering may help reduce the compensation of phosphorous at high concentrations and obtain highly efficient *n*-type diamond, still a major issue for electronic applications.

## Methods

### Ion implantation and annealing

Three high purity “quantum grade” (100) CVD single crystal diamonds from Element 6 (with [B] < 1 ppb and [N] < 5 ppb) were used for this work. The strong improve of the NV creation yield in donor-doped diamond was found in all samples (see supplementary Figures 1, 2, 3, and 4 for samples details). The ion implantations were performed using a 100 kV accelerator equipped with a sputter source, an isotopic mass selection, a beam collimation system through movable apertures of different sizes, an (x,y,z) sample stage and an optical microscope. The energies of the different ions were chosen to obtain an average implantation depth of 50 nm (28 keV, 40 keV, and 50 keV for C, N, and Mg) and 25 nm (80 keV) for Sn. These depths are low enough to ensure a little ion straggling (below 10 nm, as necessary for high-resolution implantation) and large enough to reduce the influence of the diamond surface. For the NV centres, the results are presented for the annealing temperature of 800 °C (4 h) in order to compare with previous measurements and literature. The temperature of 800 °C is generally considered as high enough to enable optimal diffusion of the vacancies to reach neighbouring nitrogen atoms and form NV centres efficiently. For the SnV and MgV centres, the results are presented for the last annealing step at 1200 °C (8 h). The full spectral temperature evolution can be found in the supplementary Figure 9.

### Donor and acceptor doping

The doping of the sample was achieved prior to the colour centre creation. Areas of different doping types and levels were prepared using ion implantation. Phosphorous (deep donor *E*_D_ = 0.57 eV) and boron (acceptor with *E*_A_ = 0.37 eV) were used in samples 1 and 2. Phosphorous, oxygen and sulphur in sample 3. The unimplanted diamond area is defined as the intrinsic area. For each dopant, a multiple implantation at different energies and fluences was conducted in order to obtain homogeneous depth concentration profiles of ~2.5 × 10^18^ cm^−3^ at the depth of 50 nm, with a thickness of ~ 50 nm (see supplementary Figures 2, 3, and 4). The dopant profiles were simulated with SRIM. The samples were then thermally annealed for four hours in vacuum in order to “activate” the dopant atoms as donors or acceptors, respectively (samples 1 and 2 at 1600 °C, sample 3 at 1200 °C). The thermal treatment also enables to heal most of the implantation defects. After annealing, the diamonds were exposed to a short oxygen plasma to clean the surface from possible graphite residuals. The samples were then implanted with different atoms for the colour centre screening study.

### Optical imaging and spectroscopy and spin measurements

The optical fluorescence imaging and spectroscopic characterisations were conducted on a home-made scanning confocal fluorescence microscope, with an air (× 100, NA = 0.95) or oil immersion (× 60, NA = 1.35) objective and two possible laser excitations at 532 nm and 488 nm. The laser reflection was suppressed by a suitable Notch filter and different spectral filters were used to select the wished fluorescence bands of neutral vacancies V^0^ (GR1 centre, with ZPL at 741 nm), NV centres (ZPL of NV^0^ at 575 nm, ZPL of NV^−^ at 638 nm), SnV centres (ZPL at 620 nm) and MgV centres (ZPL at 557 nm). The T_2_ and T_2_* spin coherence time of the single NV^−^ centres were measured on the same setup. Ramsey and Hahn echo sequences were conducted, with microwave pulses applied using a thin wire located close to the implanted centres.

### Determination of the creation yield

The creation yield of the different centres was determined in two different ways, depending on their density. If the centres could be resolved (typically for densities below 3–4 centres per µm²) they were simply counted within a reference area, and compared with the density (fluence) of implanted ions. For higher colour centres densities, single centres were taken as reference and scaled to the dense implanted areas (see supplementary information), taking background fluorescence into account.

## Supplementary information

Supplementary Information

## Data Availability

The data that support the findings of this study are available from the corresponding author upon reasonable request.
