# Supplementary material for: Coulomb-driven single defect engineering for scalable qubits and spin sensors in diamond
Source: Nat Commun. 2019 Oct 31;10:4956. doi: 10.1038/s41467-019-12556-0 (PMC6823384; doi:10.1038/s41467-019-12556-0)
Supplement: Supplementary file 1 — Supplementary Information [file 41467_2019_12556_MOESM1_ESM.pdf]

Supplementary information

Coulomb-driven single defect engineering for scalable qubits and spin sensors in diamond

T. Lühmann et al.

Supplementary information

Coulomb-driven single defect engineering for scalable qubits and spin sensors in diamond

Tobias Lühmann, Roger John, Ralf Wunderlich, Jan Meijer and Sébastien Pezzagna
Applied Quantum Systems, Felix-Bloch Institute for Solid-State Physics, University Leipzig, Germany

Supplementary methods

Samples

Three samples of the same type have been used in this work: “electronic grade” IIa CVD diamonds from element 6, with (001) polished surface and (110) facets. The impurity concentrations specified by the producer are: [N] < 5 ppb and [B] < 1 ppb. The hydrogen content is not given but it is expected to be about 1 ppm or more.
The first sample was used to study the effect of boron and phosphorous at different doping levels (3×10^{17} cm^{-3} and 3×10^{18} cm^{-3}) with respect to intrinsic diamond. The thermal annealing of the dopants (prior to the NV centres implantation) was done at 1600°C. For sample 2, the doping level of B and P was set to about 2×10^{18} cm^{-3}, using the same dopant annealing temperature of 1600°C, and different colour centres were created: NV, SnV and MgV centres. For sample 3, different donors were used (phosphorous, oxygen and Sulphur) with concentrations of 1×10^{18} cm^{-3} and pre-annealed at a lower temperature of 1200°C before the NV centres were implanted. It is known from phosphorous-doped CVD diamond that the compensation of the phosphorous donors increases with the temperature of a thermal post-treatment and that a full compensation occurs at 1600°C. The lower temperature treatment of sample 3 (1200°C) may therefore lead to less compensated donors than for samples 1 and 2. The NV creation yield in sample 3 is indeed larger than in samples 1 and 2.

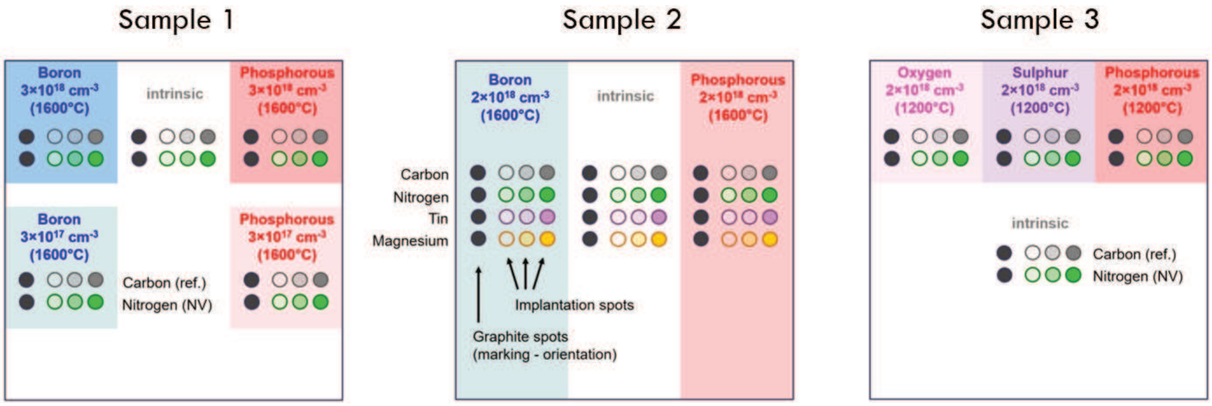

Supplementary Figure 1 | Samples. Schemes of the three samples used in this work. The pre-doped regions are indicated by the blueish zones (acceptors) and reddish zones (donors). The fluence of the different ion implantations is encoded in the colour intensity. Not at scale.

Donor and acceptor doping

Prior to the implantation of the NV, SnV and MgV centres, the different doped areas were prepared by ion implantation of acceptor (boron) and donors (phosphorous, oxygen and sulphur), followed by a 4 hours thermal annealing in vacuum at 1600°C (samples 1 and 2) and 8 hours at 1200°C (sample 3). This process enables the placement of the dopants in substitutional sites and the removal of most of the implantation-induced defects. The depth profiles of the dopants for the three samples are shown

in Figures 2, 3 and 4 (simulated using the SRIM code [1]). The ion energies and fluences were chosen to obtain homogeneous doping levels within a thickness of about 50 nm, centered at the depth of 50 nm. The details of the multiple implantations are given in the next tables.

Phosphorous		
Energy (keV)	Fluence (cm^{-2})	Fluence (cm^{-2})
30	3.4×10^{11}	3.4×10^{12}
50	5.1×10^{11}	5.1×10^{12}
90	1.3×10^{12}	1.3×10^{13}

Boron		
Energy (keV)	Fluence (cm^{-2})	Fluence (cm^{-2})
12	5.6×10^{11}	5.6×10^{12}
25	6.4×10^{11}	6.4×10^{12}
40	1.1×10^{12}	1.1×10^{13}

Supplementary Table 1 | Phosphorous and Boron implantation parameters (sample 1).

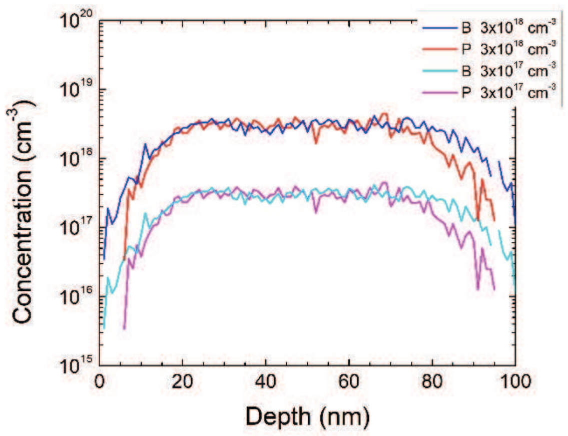

Supplementary Figure 2 | Boron and Phosphorous depth profiles (sample 1). SRIM simulation of the dopant depth profiles (see Table 1).

Phosphorous	
Energy (keV)	Fluence (cm^{-2})
30	2×10^{12}
50	2×10^{12}
70	6×10^{12}
90	6×10^{12}

Supplementary Table 2 | Phosphorous implantation parameters (sample 2).

Boron	
Energy (keV)	Fluence (cm^{-2})
15	3×10^{12}
25	5×10^{12}
40	8×10^{12}

Supplementary Table 3 | Boron implantation parameters (sample 2).

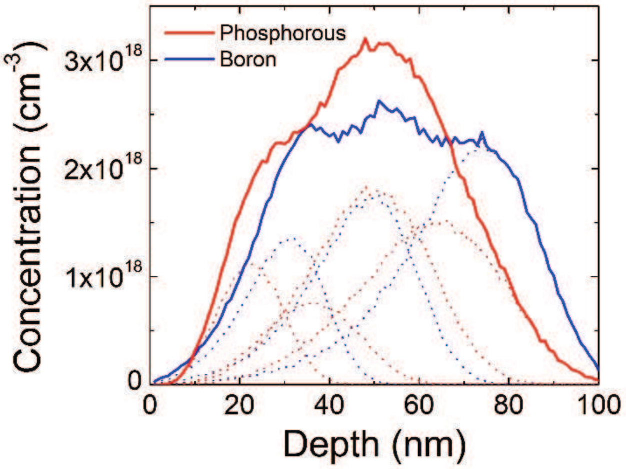

Supplementary Figure 3 | Boron and Phosphorous depth profiles (sample 2). SRIM simulation of the dopant depth profiles. The dotted lines represent the implantations done at different ion energies and fluences (Tables 2 and 3) to obtain a homogeneous doping level on a 50 nm thickness.

Phosphorous		Oxygen		Sulphur	
Energy (keV)	Fluence (cm^{-2})	Energy (keV)	Fluence (cm^{-2})	Energy (keV)	Fluence (cm^{-2})
40	4.1×10^{12}	25	2.1×10^{12}	40	1.6×10^{12}
80	4.1×10^{12}	50	4.2×10^{12}	80	3.7×10^{12}

Supplementary Table 4 | Phosphorous, oxygen and sulphur implantation parameters (sample 3).

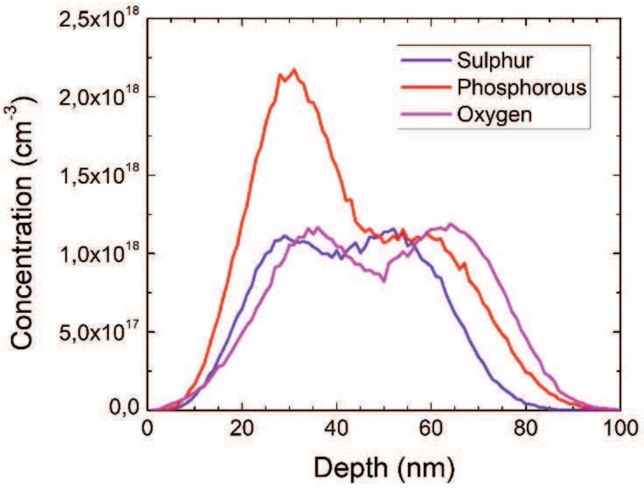

Supplementary Figure 4 | Sulphur, Phosphorous and Oxygen depth profiles. SRIM simulation of the dopant depth profiles (see Table 4).

Ion implantation – Mass spectra

The ion species used in this work were produced by a 100 kV ion accelerator equipped with a cesium sputter source. Cathodes of different elements are self-made and enable the extraction of a large number of chemical elements. The ions produced by this means are negatively charged. The mass selection is done by a 90° bending magnet. The corresponding mass spectra used to produce NV, MgV and SnV centres are shown in Figure 3.

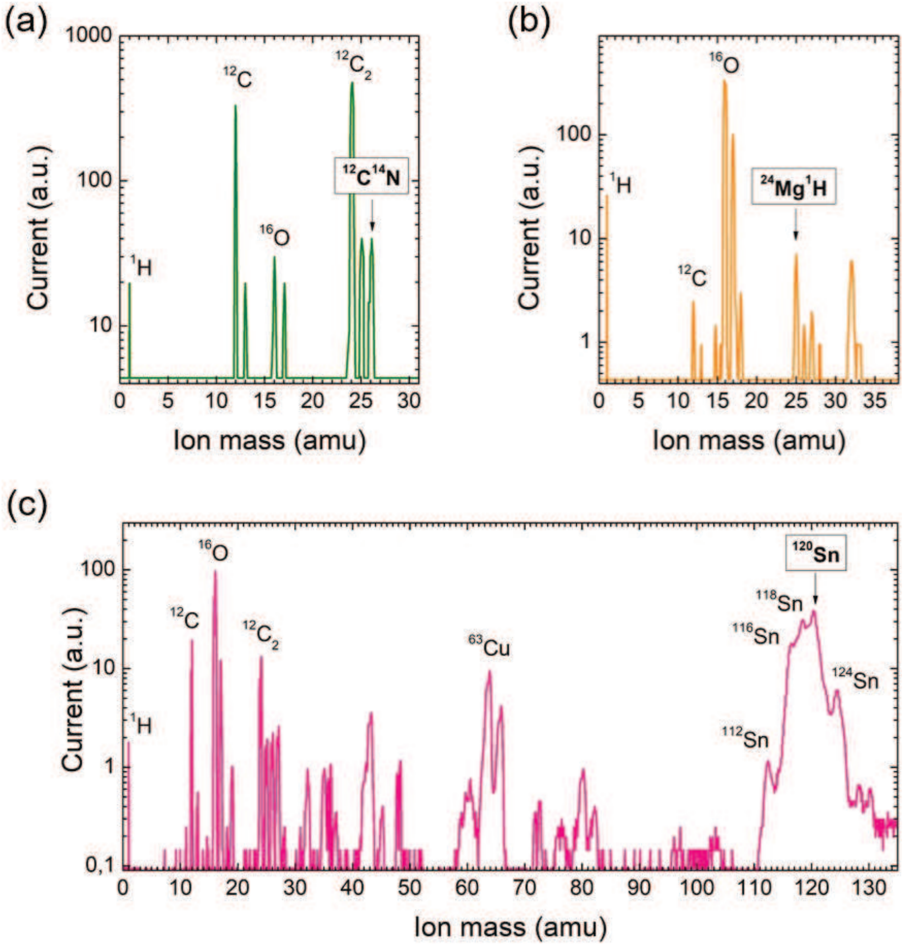

Supplementary Figure 5 | Mass spectra. (a) The molecular ion $(^{12}C^{14}N)^{-}$ was used to produce NV centres. (b) The ion $(^{24}Mg^{1}H)^{-}$ was used to produce MgV centres. Despite the co-implantation of a hydrogen (which is anyway found in high concentration within the diamond) the use of this mass 25 has avoids the ambiguity of implantation of C_2. Furthermore, it is almost impossible to produce Mg^{-}. (c) The ion $^{120}Sn^{-}$ was used for the creation of SnV centres, from a SnPb cathode.

Ion implantation – Depth and of NV, SnV and MgV centres

The depth profiles of the different elements used to produce colour centres are plotted in Figure 6. The corresponding acceleration energies are given in the inset.

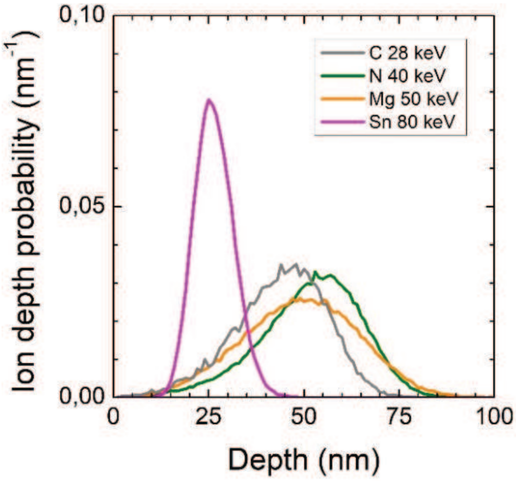

Supplementary Figure 6 | Implantation depth of NV, SnV and MgV centres simulated using SRIM.

Note that in the case of tin, the average depth is about 25 nm instead of 50 nm for the other elements. This is due to technical limitations of the accelerator voltage and to the large mass of tin. The phosphorous and boron doping are therefore about one third weaker than for the other elements, as it can be seen in Figure 3 (doping depth profiles).
More precisely, [B] ~ 1.6×10^{18} cm^{-3} and [P] ~ 2.0×10^{18} cm^{-3} for tin, whereas [B] ~ 2.3×10^{18} cm^{-3} and [P] ~ 3.0×10^{18} cm^{-3} for carbon, nitrogen and magnesium.

Determination of the creation yield of colour centres

The method to determine the creation yield of the implanted colour centres depends on their density: whether or not it is possible to optically separate them with the fluorescence confocal microscope. If the centres density is less than typically 3 – 4 centres / µm², then one simply counts the number of centres per unit area and compare it to the ion implantation fluence. If the centres density exceeds this value then the yield is determined as shown in figure 4.
A small scan is recorded at the edge of the implantation spot, where the lower centres density enables the observation of single centres that can be used as reference for the fluorescence intensity. A reference is also taken from an unimplanted area to obtain the background fluorescence.

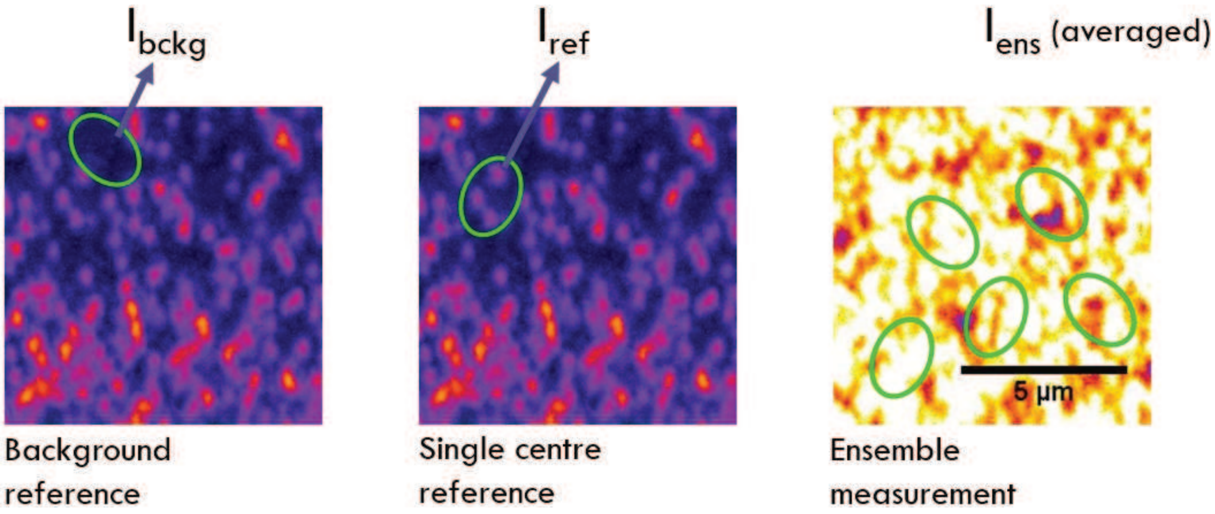

Supplementary Figure 7 | Determination of the creation yield of colour centres.

First, a background reference is done by integrating the fluorescence intensity of an area of surface S, free of centres, giving I_{bckg}.
Then, a single centre reference is calculated by integrating the fluorescence intensity of an area of surface S, containing a well-defined number Q_{NVs} of isolated and single centres, giving I_{ref}.
The reference value for a single centre is given by:

$$I_{\mathrm{single}} = \frac{I_{\mathrm{ref}} - I_{\mathrm{bckg}}}{Q_{\mathrm{NVs}}} \tag{1}$$

The density of created centres is obtained from the integration over a surface S of the fluorescence intensity within the implantation spot I_{ens}:

$$\text{Centres' density} = \frac{1}{S} \times \frac{I_{\mathrm{ens}} - I_{\mathrm{bckg}}}{I_{\mathrm{single}}} \tag{2}$$

Finally, the creation yield is given by the ratio of centres' density and implanted ion fluence F:

$$\text{Creation Yield} = \frac{\text{Centres' density}}{\text{ion fluence}} = \frac{Q_{\mathrm{NVs}}}{S\,F}\left(\frac{I_{\mathrm{ens}} - I_{\mathrm{bckg}}}{I_{\mathrm{ref}} - I_{\mathrm{bckg}}}\right) \tag{3}$$

Supplementary discussion

Single MgV centre

The absorption of single Mg-related centres reveals a strong polarisation anisotropy (Figure 8) which likely indicates the involvement of vacancy in the centre. This polarisation-dependent absorption is strongest along the [110] axis (for a sample with a (100) surface).

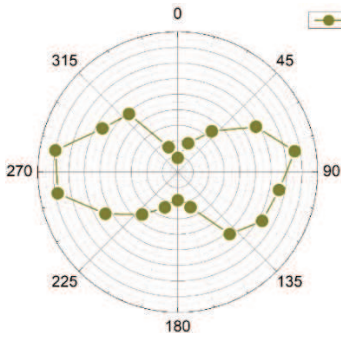

Supplementary Figure 8 | Polarisation-dependent absorption of a single MgV centre.

Spectral evolution with temperature

The optical signatures of NV^0 (ZPL at 575 nm) and NV^- (ZPL at 638 nm) centres are well known. The evolution of the emission spectra of NV ensembles with temperature is strongly modified between 600°C and 800°C (Figure 9a). This is due to the annealing out of vacancies which are deeper acceptors than NV centres. Therefore, the NVs are less NV^- at 600°C when a large amount of vacancies is still present, whereas they are more NV^- at 800°C when the vacancies have been mostly annealed out. This is clearly evidenced in Figure 10 which plots the difference of the (normalised) emission spectra at the two temperatures.

On the other hand, the fluorescence spectra of the MgV centres do not change (at least in the detection window 550 nm – 900 nm) neither on the whole temperature range, nor in the different doping areas, at the exception of the weak peak at 544.5 nm. It is at this stage impossible to conclude about charge states of MgV centres.

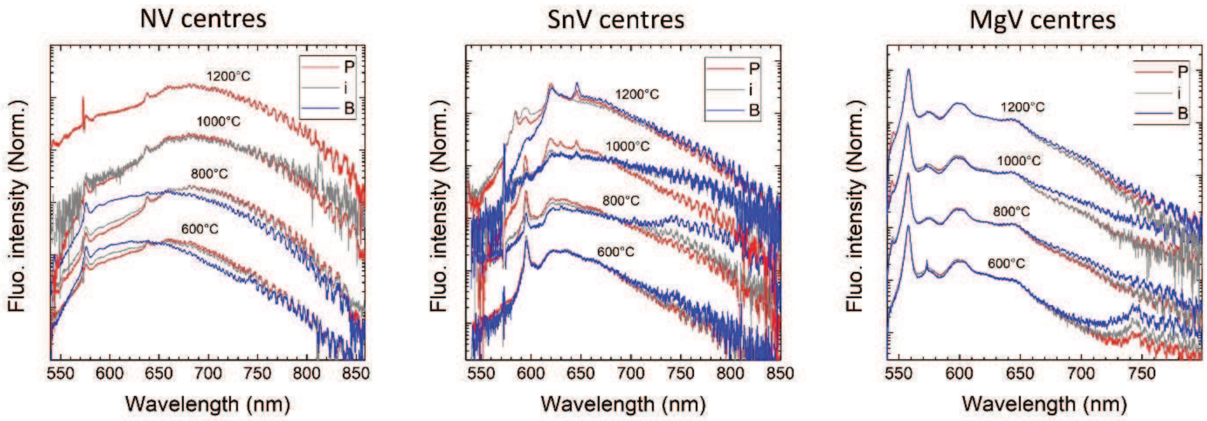

Supplementary Figure 9 | Fluorescence spectra (normalised and separately plotted for clarity) of ensembles of NV, SnV and MgV centres as a function of temperature and diamond doping.

Finally, the SnV centres present a complex evolution of emission features with temperature. Due to the large mass of Sn, much higher temperatures than 1200°C are required to obtain proper emission spectra of SnV centres. This was shown in reference [2]. With the results of this study, that is the strongly doping-dependent creation of defects in diamond, it is difficult to attribute charge states or

Sn-related forms to the different optical signatures. Note that the SnV^- charge state is attributed to the 620 nm ZPL [3] and the SnV^0 was calculated to be at 682 nm [4], which is not observed here, even in boron doped diamond. This is likely due to the too low annealing temperature (see reference [2]).

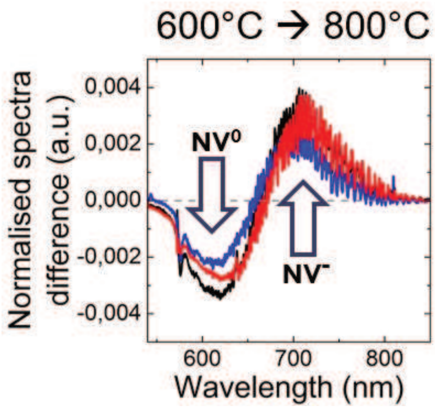

Supplementary Figure 10 | Evidence for the acceptor behaviour of the vacancies. Difference of fluorescence spectra (normalised) of ensembles of NV (in Phosphorous (red), intrinsic (black) and Boron (blue) areas) between the two temperature steps of 600°C and 800°C. The shift towards NV- when the temperature is increased is the signature of the annealing out of the vacancies which act as acceptors.

Cathodoluminescence measurement

In Figure 2a of the main manuscript, bright dots are visible in some of the implanted spots. These dots are due to cathodoluminescence (CL) experiments conducted in the carbon implanted diamond areas (n-type, intrinsic and p-type) prior to annealing in order to check whether the signal from vacancies can be detected in CL. The sample was cooled down to 77K and the electron beam energy set to 5 keV. Figure 11 shows the CL spectra recorded in the different doped areas. The ZPL at 741 nm of the neutral isolated vacancies can be recognised. It can be seen that, as already measured in PL, the V^0 fluorescence is quasi-absent in the phosphorous doped region where the vacancies are negatively charged.

It is not fully understood why an additional GR1 signal is induced by the electron beam. It may be due either to a charging effect modifying the charge state balance within the sample, thus revealing the native vacancies, or to an unwanted ion implantation if a negative potential builds up at the diamond surface (leading to the implantation of species from the rest gas within the chamber which are ionised by the electron beam and accelerated to the surface, as already shown in [5]).

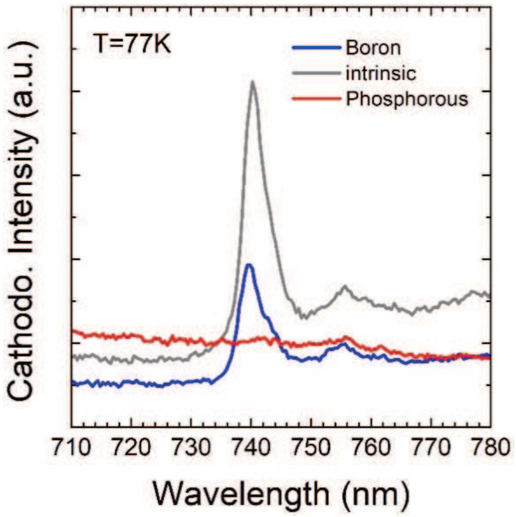

Supplementary Figure 11 | Cathodoluminescense spectra at T=77K of the carbon implanted spots (fluence 2×10^{13} cm^{-2}) within the three different doped areas prior to thermal annealing. The electron beam energy was set to 5 keV. The GR1 fluorescence peak at 741 nm from the neutral isolated vacancies is visible in the intrinsic and boron-doped areas whereas it is not present in the phosphorous-doped region because the vacancies are negatively charged.

Supplementary references

[1] Ziegler J. The stopping and range of ions in matter, *SRIM-2008, online at* http://srim.org (2008).

[2] Iwasaki T. *et al.* Tin-vacancy quantum emitters in diamond, *Phys. Rev. Lett.* **119**, 253601 (2017).

[3] Thiering G. & Gali A. Ab initio magneto-optical spectrum of group-IV vacancy color centers in diamond, *Phys. Rev. X* **8**, 021063 (2018).

[4] Thiering G. & Gali A. The $(e_g \otimes e_u) \otimes E_g$ product Jahn-Teller effect in the neutral group-IV – vacancy quantum bits in diamond, *arXiv:1807.1063v2* (2019).

[5] Becker S., Raatz N., Jankuhn., John R. & Meijer J. Nitrogen implantation with a scanning electron microscope, *Scientific Reports* **8**, 32 (2018).
